# The Role of Polyphenoloxidase, Peroxidase, and β-Glucosidase in Phenolics Accumulation in *Olea europaea* L. Fruits under Different Water Regimes

**DOI:** 10.3389/fpls.2017.00717

**Published:** 2017-05-09

**Authors:** Marco Cirilli, Giovanni Caruso, Clizia Gennai, Stefania Urbani, Eleonora Frioni, Maurizio Ruzzi, Maurizio Servili, Riccardo Gucci, Elia Poerio, Rosario Muleo

**Affiliations:** ^1^Laboratorio di Ecofisiologia Molecolare delle Piante Arboree, Dipartimento di Scienze Agrarie e Forestali, Università degli Studi della TusciaViterbo, Italy; ^2^Dipartimento di Scienze Agrarie, Alimentari e Agro-ambientali, Università di PisaPisa, Italy; ^3^Dipartimento di Scienze Agrarie, Alimentari ed Ambientali, Università degli studi di PerugiaPerugia, Italy; ^4^Dipartimento per la Innovazione nei Sistemi Biologici, Agro-alimentari e Forestali, Università degli Studi della TusciaViterbo, Italy; ^5^Tree and Timber Institute, National Research Council of ItalySesto Fiorentino, Italy

**Keywords:** enzyme activities, phenols, secoiridoids, catabolism, olive, oleuropein, relative transcript level, water deficit

## Abstract

Olive fruits and oils contain an array of compounds that contribute to their sensory and nutritional properties. Phenolic compounds in virgin oil and olive-derived products have been proven to be highly beneficial for human health, eliciting increasing attention from the food industry and consumers. Although phenolic compounds in olive fruit and oil have been extensively investigated, allowing the identification of the main classes of metabolites and their accumulation patterns, knowledge of the molecular and biochemical mechanisms regulating phenolic metabolism remains scarce. We focused on the role of polyphenoloxidase (*PPO*), peroxidase (*PRX*) and β-glucosidase (β-*GLU*) gene families and their enzyme activities in the accumulation of phenolic compounds during olive fruit development (35–146 days after full bloom), under either full irrigation (FI) or rain-fed (RF) conditions. The irrigation regime affected yield, maturation index, mesocarp oil content, fruit size, and pulp-to-pit ratio. Accumulation of fruit phenolics was higher in RF drupes than in FI ones. Members of each gene family were developmentally regulated, affected by water regime, and their transcript levels were correlated with the respective enzyme activities. During the early phase of drupe growth (35–43 days after full bloom), phenolic composition appeared to be linked to β-GLU and PRX activities, probably through their effects on oleuropein catabolism. Interestingly, a higher β-GLU activity was measured in immature RF drupes, as well as a higher content of the oleuropein derivate 3,4-DHPEA-EDA and verbascoside. Activity of PPO enzymes was slightly affected by the water status of trees during ripening (from 120 days after full bloom), but was not correlated with phenolics content. Overall, the main changes in phenolics content appeared soon after the supply of irrigation water and remained thereafter almost unchanged until maturity, despite fruit growth and the progressive decrease in pre-dawn leaf water potential. We suggest that enzymes involved in phenolic catabolism in the olive fruit have a differential sensitivity to soil water availability depending on fruit developmental stage.

## Introduction

Over the last 20 years, the world consumption of olive oil has increased by 54% (International Olive Council [IOC], 2016), mostly due to a growing consumption in countries outside of the Mediterranean region. An increasing body of evidence suggests that the beneficial effects of VOO arise not only from its balanced fatty acid composition, but also from the presence of bio-active minor components such as tocopherols and phenolic compounds. Olive phenolic compounds have antioxidant properties and affect organoleptic properties of the olive fruit and oil.

The phenolics composition of olive fruits and derived VOOs is affected by many factors, namely cultivar, fruit development, climate conditions, and cultural practices (Ryan and Robards, 1998; Servili et al., 2007a; Tura et al., 2008). Several studies have focused on changes in the phenolics composition and content during fruit development until ripening (Amiot et al., 1986; Ryan et al., 1999; Alagna et al., 2012; Talhaoui et al., 2015). Secoiridoids, which include oleuropein, ligstroside, and their aglycon derivates *p*-HPEA-EA (ligstroside aglycon) and 3,4-DHPEA-EA (oleuropein aglycon isomer), dialdehydic forms of elenolic acid (3,4-DHPEA-EDA and *p*-HPEA-EDA), phenylethanoids such as tyrosol (*p*-HPEA) and hydroxytyrosol (3,4-DHPEA), and verbascoside (a phenylpropanoid) are the main phenols in olive fruit, whereas flavonoids (rutin, luteolin, and cyanidin) represent only a minor fraction (Servili et al., 2004). The total phenol content is highest in immature drupes and gradually decreases during fruit development, although the rate of change varies depending on cultivar and environmental conditions (Monteleone et al., 1995; Romani et al., 1999; Rotondi et al., 2004; Talhaoui et al., 2015). Oleuropein and its derivative 3,4-DHPEA-EDA are the main bisphenols in olive mesocarp (Amiot et al., 1986; Alagna et al., 2012). During fruit growth, oleuropein progressively decreases concomitantly with the increase of 3,4-DHPEA-EDA. Some cultivars are also able to synthesize demethyloleuropein, which increases during ripening (Sivakumar et al., 2007; Alagna et al., 2012). Other secoiridoids, such as ligstroside (*p*-HPEA-EDA), hydroxytyrosol (3,4-DHPEA), and tyrosol (*p*-HPEA) follow the same decreasing trend as oleuropein during fruit maturation and ripening (Servili et al., 1999, 2004; Morelló et al., 2004), whereas the verbascoside content does not follow an unequivocal pattern (Amiot et al., 1986; Ryan et al., 2002; Alagna et al., 2012). Despite this knowledge, molecular and biochemical mechanisms regulating the accumulation of specific phenolic metabolites in the olive fruit are still far from being clear. Candidate genes involved in the biosynthesis of secoiridoids, phenylpropanoids, and flavonoids have recently been identified in *Olea europaea* (Alagna et al., 2009; Iaria et al., 2016). Nevertheless, several steps in the secoiridoid biosynthetic pathways remain unknown, as well as the general role of specific enzymatic classes, including the polyphenoloxidase (PPO), peroxidase (PRX), and β-glycosidase (β-GLU) families. The quantity and localization of in different fruit tissues affect the phenolic composition and oil quality of VOO during extraction (Luaces et al., 2007; Servili et al., 2007a). Endogenous glycosidase and esterase activities were hypothesized to play a pivotal role in the regulation of oleuropein hydrolysis (Amiot et al., 1989; Gutierrez-Rosales et al., 2010, 2012). Recently, several members of the β-glucosidase family have been found in proteomic and transcriptomic studies (Corrado et al., 2012; Bianco et al., 2013). An olive β-glucosidase (Oeβ-GLU) able to deglycosilate oleuropein with high affinity has recently been isolated and functionally characterized (Koudounas et al., 2015).

Water availability represents the main limiting factor for growth and yield of crops of the Mediterranean region. A wide array of physiological responses, signals and genes are triggered in response to drought (Shao et al., 2008; Krasensky and Jonak, 2012). When the water stress signal reaches a threshold value, it determines morphological and physiological changes, including the production of reactive oxygen species (ROS) that act as an alarm to induce plant survival responses (Cruz de Carvalho, 2008). As phenolic compounds have strong antioxidant properties, a role in the protection against ROS during water stress adaptation has been long suggested (Ramakrishna and Ravishankar, 2011). Olive trees are typically adapted to long periods of high temperatures and drought during the summer (Lavee et al., 1991). Soil water availability dramatically affects the concentration of phenolic compounds during fruit development (Servili et al., 2007b; Caruso et al., 2014). In Greek cultivars, severe water stress induced an increase in total phenol content, mainly due to a rise in oleuropein content (Petridis et al., 2012). A positive relationship between total phenol content and antioxidant activity has also been detected, suggesting that phenols could play a relevant role in the protection against the effects of drought (Petridis et al., 2012). The effect of three different irrigation water regimes on analytical parameters of olive oil was evaluated in cultivars ‘Leccino’ and ‘Frantoio’ (Servili et al., 2007b; Caruso et al., 2014). Full irrigation (FI) decreased the concentration of total phenol and *o*-diphenol in VOOs, with wide differences in the concentration of the aglycone derivate of oleuropein. In olive trees of cultivar ‘Arbequina’ subjected to four irrigation management approaches, at different stages of fruit development, the highest content of total phenols, hydroxytyrosol acetate, 3,4-DHPEA-EDA, *p*-HPEA-EDA, 3,4-DHPEA-EA, *o*-diphenols, tyrosyl elenolate (*p*-HPEA-EA), and total secoiridoids was detected in trees stressed from the end of fruit drop to the end of July (Del Campo and García, 2013). Fruit ripening and irrigation treatments have been also found to induce considerable variation in the concentrations of secoiridoid derivatives of hydroxytyrosol and tyrosol in VOO of cultivars ‘Cornicabra’ (Gümez-Rico et al., 2006), ‘Souri’ (Dag et al., 2008), and ‘Cipressino’ (Martinelli et al., 2013). Similar results were found in trees of cultivar ‘Leccino,’ where the highest content of phenolic compounds was detected in rain-fed (RF) trees, although the transcript level of the *PAL* gene did not differ among the water regimes (Martinelli et al., 2012).

Since there are no reports providing molecular evidence concerning the role of gene families involved in the oxidative catabolism of phenolic compounds in olive fruit, we set up a field experiment to provide insight about the role of PPOs, PRXs, and β-GLUs in the accumulation of phenolic compounds in olive fruits during their development and ripening. We used an integrated approach at the molecular, biochemical, and metabolic levels to investigate the role of the above enzymes on phenolic metabolism in fruits when the water status was manipulated by imposing either FI or RF conditions to field-grown olive trees (‘Frantoio’).

## Materials and Methods

### Plant Material and Site Characteristics

Experiments were conducted in a fully productive, irrigated olive (‘Frantoio’) orchard located at Venturina, Italy, in 2011. Olive trees had been planted at a spacing of 5 m × 3.9 m in April 2003. The soil was a deep (1.5 m), sandy-loam (ISSS classification), consisting of 60% sand, 15% clay, and 25% silt. The climate at the study site was sub-humid Mediterranean (Caruso et al., 2013), with an annual mean temperature and annual rainfall of 15°C and 635 mm, respectively (means of 21 years, 1990–2010). Climatic conditions over the study period were monitored using an iMETOS IMT 300 weather station (Pessl Instruments GmbH, Weiz, Austria). Reference annual evapotranspiration (ET_0_), calculated according to the Penman–Monteith equation, was 840 mm. The year 2011 was hot and dry with annual and summer (21 June–22 September) precipitations of 197 and 18 mm, respectively (Supplementary Figure S1). Cultural practices and monitoring of phenological parameters were performed as previously reported (Caruso et al., 2013). In 2011, full bloom, estimated as when 70% of inflorescences showed at least 50% of flowers open, occurred on 24 May. Three blocks, each consisting of two irrigation treatments (three plots per treatment) randomly distributed, were used for the trial. Each of the six plots included 12 trees arranged in three rows of four trees. Only the inner trees of the central row were used for monitoring the tree water status, and only four of the six trees per treatment were used for measurements and sampling.

### Irrigation and Tree Water Status

Subsurface drip irrigation lines (2.3 L h^-1^ pressure-compensated drippers spaced at 0.6 m), placed at a depth of 0.35–0.40 m and 0.8 m distance from the tree row, were used to supply 100% (FI) or 2% (RF) of water requirements calculated from reference evapotranspiration using a crop coefficient of 0.55 (Caruso et al., 2013). RF trees received a total of 33 m^3^ ha^-1^ irrigation on three dates [93, 94, and 113 days after full bloom (DAFB)], to avoid tree damage due to extreme water stress. Therefore, the RF condition was partially alleviated by three complementary irrigations because of the particularly dry year. The irrigation period lasted from 1st July to 26th September, and FI trees received water 4–5 days a week (3–7 h per day); the volume applied was 734 m^3^ ha^-1^, corresponding to 1431 L per tree. Irrigation volumes were calculated on the basis of the effective evapotranspiration, and tree water status was determined by measuring pre-dawn leaf water potential (PLWP) during the dry season at 7–10 days intervals. Leaves were excised with a razor blade, immediately put in the chamber cylinder (Tecnogas, Pisa, Italy), which was then pressurized with nitrogen gas at a maximum rate of 0.02 MPa s^-1^ (Caruso et al., 2013). Fertigation was used to supply mineral nutrients in spring, before irrigation treatments were put into action. Each tree received a total of approx. 90 g of N, P_2_O_5_, and K_2_O.

### Fruit Growth and Production

Five fruits per tree in the south-east sector of the canopy were identified prior to the beginning of irrigation and their volume measured non-destructively by water displacement using a graduated cylinder. Fruits were sampled for enzymatic studies and determination of phenolic compounds at 35, 43, 63, 77, 93, 115, 136, and 146 DAFB and immediately frozen in liquid nitrogen. Frozen samples were finely ground in liquid nitrogen using a mortar and pestle and preserved at -80°C until biochemical and molecular analyses was carried out. The number of fruits sampled at each date was adjusted to account for fruit growth and obtain sufficient material for further analysis. In particular, 20 (35 DAFB), 15 (43, 63, and 77 DAFB), and 10 (93, 115, 136, and 146 DAFB) fruits were sampled from each of the four trees per treatment. Immediately before harvest, which occurred 146 DAFB, 50 fruits were randomly sampled from around the canopy of each tree to measure average fruit weight. The same fruits were also scored based on the color of the exocarp and mesocarp using a 0–7 arbitrary scale to determine the maturation index (MI) according to standard methodology (Caruso et al., 2013). Each tree was harvested individually by hand. The total number of fruits per tree was calculated by dividing the crop yield by the average fruit weight. At harvest (146 DAFB), five fruits per tree, similar to those used for enzyme assays and phenolic concentrations, were destructively sampled and their fresh weight (FW) determined. The mesocarp was separated from the endocarp using a sharp blade, the FW of both tissues was measured, and then the dry weight (DW) was determined after oven drying at 70°C to constant weight. The oil content of the fruit mesocarp of five fruits per tree, previously sampled for fresh and dry weight determinations, was also measured at harvest by nuclear magnetic resonance using an Oxford MQC-23 analyser (Oxford Analytical Instruments Ltd., Oxford, UK) as previously reported by Caruso et al. (2013).

### High-Performance Liquid Chromatography (HPLC) Analysis of Phenolic Compounds

Fruits samples were frozen in liquid nitrogen and stored at -80°C, and successively used for phenolic determination. The phenols were extracted from the olive pulp according to the procedure described by Servili et al. (2012) modified as follows: 5 g of frozen olive pulp was homogenized with 100 mL of 80% methanol containing 20 mg L^-1^ butylated hydroxytoluene (BHT); the extraction was performed in triplicate. After methanol removal, the aqueous extract was used for extraction by solid-phase separation (SPE) of phenols. The SPE procedure was applied by loading a 1000 mg Bond Elute Jr-C18 cartridge (Agilent Technologies, Santa Clara, CA, USA) with 1 mL of sample, using 50 mL of methanol as the eluting solvent. After solvent removal under vacuum at 30°C, the phenolic extract was recovered and then dissolved in methanol (1 mL) and filtered through a polyvinylidene fluoride (PVDF) syringe filter (0.2 μm). The HPLC analyses of the phenolic extracts were conducted according to the method of Selvaggini et al. (2006) with a reversed-phase column using an Agilent Technologies system Model 1100 (Agilent Technologies, Santa Clara, CA, USA) that was composed of a vacuum degasser, a quaternary pump, an autosampler, a thermostatted column compartment, a diode array detector (DAD), and a fluorescence detector (FLD). The C18 column used in this study was a Spherisorb ODS-1 250 mm × 4.6 mm with a particle size of 5 μm (Waters, Milford, MA, USA); the injected sample volume was 20 μL. The mobile phase was composed of 0.2% acetic acid (pH 3.1) in water (solvent A)/methanol (solvent B) at a flow rate of 1 mL min^-1^, and the gradient was changed as follows: 95% A/5% B for 2 min, 75% A/25% B over 8 min, 60% A/40% B over 10 min, 50% A/50% B over 16 min, and 0% A/100% B over 14 min; this composition was maintained for 10 min, then returned to the initial conditions and equilibration over 13 min; the total running time was 73 min. Lignans were detected by an FLD operated at an excitation wavelength of 280 nm and emission at 339 nm, while the other compounds were detected by DAD at 278 nm.

### HPLC Analysis of VOO Phenolic Compounds

The extraction of VOO phenolic compounds was performed as reported by Montedoro et al. (1992). The HPLC analyses of the phenolic extracts were conducted according to the method of Selvaggini et al. (2014); for the detection of phenolic compounds, a DAD was employed with the wavelength set at 278 nm.

### Enzyme Extraction and Activities

Two hundred milligrams of fruit pulp frozen powder was suspended in 1 mL of an extraction buffer consisting of 50 mM potassium phosphate, 1 mM EDTA, 1 mM PMSF, and 1% (w/v) PEG4000, pH 6.2. The suspension was shaken at 2000 rpm for 1 h at 4°C, and the supernatant was recovered by centrifugation (12000 rpm for 15 min at 4°C). The pellet was re-extracted, and the two supernatants were combined, filtered through a 0.45 μm filter (Sartorius, Italy), and used for enzyme activity assays.

Polyphenoloxidase activity was measured at 25°C, according to the method of Zouari-Mechichi et al. (2006), by monitoring oxidation of 2,6-dimethoxyphenol (2,6-DMP) spectrophotometrically (𝜀_469nm_ = 27.5 mM^-1^ cm^-1^); the reaction mixture (1 mL final volume) consisted of 5 mM 2,6-DMP in McIlvaine buffer at pH 6.0. PRX activity was measured at 25°C, according to the method of Makkar et al. (2001), by monitoring oxidation of 2,2′-azino-bis(3-ethylbenzothiazoline-6-sulfonic acid) (ABTS) spectrophotometrically (𝜀_420_ = 36.0 mM^-1^ cm^-1^); the reaction mixture (1 mL final volume) consisted of 5 mM ABTS and 0.2 mM H_2_O_2_ in McIlvaine buffer at pH 3.0.

β-glycosidase activity was measured at 25°C, according to Romero-Segura et al. (2009), by monitoring formation of *p*-nitrophenol spectrophotometrically (𝜀_405_ = 0.553 mM^-1^ cm^-1^) due to hydrolysis of *p*-nitrophenyl-β-D-glucopyranoside (*p*-NPG). The reaction mixture (1 mL final volume) consisted of 5 mM *p*-NPG in McIlvaine buffer at pH 6.0. All enzyme activities were expressed as IU per g (FW) fruit tissue.

### Identification of Putative Genes Coding for Enzymes of Phenols Degradation

Sequences of transcripts coding for *PPO, PRX*, and β-*GLU* genes were identified by a tBLASTn approach, implemented in BioLign 4.0^1^, using amino acid sequences of enzymes already characterized in other plant species, including *Arabidopsis thaliana, Malus domestica* Borkh., *Vitis vinifera* L., and *Populus trichocarpa*. A search for *O. europaea* orthologous genes was performed by exploring olive fruit^2^ (Alagna et al., 2009) and flower EST databases (Alagna et al., 2016). Identified transcripts were annotated by BLAST against the NCBI-*nr* database and used as a query to retrieve genomic sequences of each gene from an advanced genome assembly of cultivar ‘Leccino’ (Olea Genome Project). Genomic sequences were aligned with EST clusters to reconstruct the full-length ORF tentative consensus. Specific primers were designed by using Primer3 software^3^ (Supplementary Table S1).

### Amino Acid Sequence Comparisons and Phylogenetic Analysis

The full predicted amino acid sequences of candidate genes were used to reconstruct the phylogeny with their homologs from others species. Alignments were performed using the ClustalW2 algorithm^4^ with default parameters and GeneDoc software. Gene models for multiple alignment analysis were obtained from the Phytozome V11.0 database^5^ (Supplementary Table S2). A rooted tree was reconstructed using the neighbor-joining method in MEGA6 software (Tamura et al., 2013). Tree nodes were evaluated by bootstrap analysis of 1500 replicates (pairwise deletion, uniform rates, and Poisson correction options). Intracellular localization was inferred for each protein by the TargetP1.1 server^6^.

### Real-Time Quantitative PCR Expression Analysis

Total RNA was extracted from fruit tissues, following the guidelines of a modified protocol of Doyle and Doyle (1990). Samples were DNase treated using an RNeasy Plant Mini Kit (Qiagen, Cat. No. 74904, Italy), following the manufacturer’s instructions. RNA purity was evaluated by agarose gel electrophoresis and quantified using a QUBIT^®^ 2.0 Fluorometer (Invitrogen, Cat. No. Q32866, Italy). First-strand cDNA was synthesized using Ready-To-GO^TM^ RT-PCR Beads (GE Healthcare^TM^ Illustra^TM^, Cat. No. 27-9267-01, Italy), following the manufacturer’s guidelines. Real-time PCR analysis was conducted using the thermal cycler LC480II^®^(Roche, Italy). Each reaction (20 μL) contained 10 μL of LightCycler 480 SYBR Green I Master (Roche, Cat. No. 04 707 516 001, Italy), 0.5 μM of each primer, 1 μL of cDNA, and 7 μL of PCR-grade water. The PCR reaction was conducted using the following conditions: 95°C for 10 min; 45 cycles at 94°C for 20 s, 59°C for 30 s, and 72°C for 30 s; followed by a melting cycle from 65–95°C. Real-time quantitative PCR was performed using three biological replicates, with three technical replicates for each sample. Data were expressed with the 2^ΔCp^ method (Kubista et al., 2006) using the geometric means of the *elongation factor-1 alpha* (*OeEF1-*α, no. AM946404.1) and actin genes as endogenous reference genes for the normalization of transcript abundance. After PCR amplification, all products were sequenced to confirm their identity.

## Results

### Water Status and Yield Components

**FIGURE 1:**
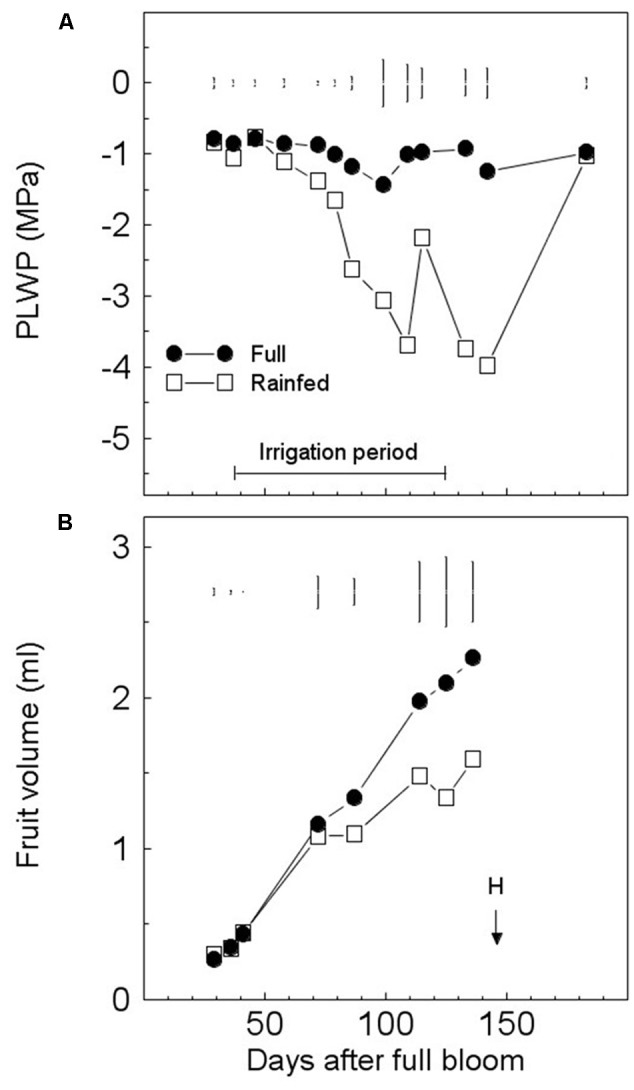
**Seasonal variations in pre-dawn leaf water potential (PLWP) (A)** and fruit growth of olive trees (‘Frantoio’) **(B)** under full (FI) or rain-fed irrigation (RI). Symbols are means of six (tree water status) or four (fruit growth) trees per treatment. Vertical bars represent least significant differences (LSD) between irrigation treatments after analysis of variance (ANOVA) (*p* < 0.05). H, harvest date.

The PLWP of the FI trees was usually maintained at approx. -1 MPa by irrigation. The PLWP dropped below -1 MPa three times and temporarily reached -1.43 MPa at 99 DAFB during the irrigation period due to pump failure (**Figure 1A**). The PLWP of RF trees decreased progressively with increasing seasonal drought and reached very low values of -3.70 and -3.99 MPa at 109 and 142 DAFB (**Figure 1A**). Fruits from FI trees grew according to an almost linear pattern (**Figure 1B**). The size of FI fruits was greater than that of RF ones starting from 87 DAFB until harvest, when fruit volume of RF fruits was only 70% that of FI fruits (**Figure 1B**). Fruit yield of FI trees was higher (227%) and significantly different from that of RF trees (**Table 1**). The number of fruits of FI trees was higher, but not significantly different, than that of RF trees, and this difference disappeared if the number of fruits per tree was expressed on a trunk cross-sectional area basis (**Table 1**). Significant differences in fruit FW were found between the two irrigation regimes; the maturation index (pigmentation of skin and pulp) was delayed in FI trees (**Table 1**). The oil yield of the RF treatment was 41% of that of FI trees.

**Table 1:** Yield, yield components, yield efficiency (fruit yield/TCSA), and maturation index (MI) of olive trees (‘Frantoio’) subjected to full irrigation (FI) or rain-fed (RF) conditions.

Irrigation	Fruit yield (g/tree)	Fruit yield/TCSA (g/dm^-2^)	Fruits/tree	Oil yield (g/tree)	Fruit FW (g)	MI	Oil in mesocarp (%/DW)
FI	8070b	3437	3255	1960b	2.5b	3.2a	68.3b
RF	3559a	2074	2311	792a	1.5a	4.0b	58.4a
*LSD*	*3339*	*1580*	*1454*	*985*	*0.2*	*0.4*	*2.0*

*Values are means of four trees per treatment (*n =* 4). Different letters indicate least significant differences (LSD) between irrigation treatments after analysis of variance (ANOVA) within each year (*p* < 0.05). TCSA, trunk cross-sectional area; FW, fresh weight; DW, dry weight.*

Trees subjected to substantial water deficit (RF) had fruit measurements significantly lower than those for FI trees for all fruit parameters, except the endocarp DW (**Table 2**). The mesocarp, endocarp, and whole fruit FW of FI trees was 182, 130, and 162% of those of the RF trees. The meso-to-endocarp ratio, the FW to DW ratio, and the mesocarp moisture were significantly higher (136, 126, and 130%, respectively) in fruits from FI trees compared with fruits from RF trees (**Table 2**).

**Table 2:** Fresh weight and dry weight of fruit, mesocarp, and endocarp, meso-to-endocarp ratio, fruit FW/DW ratio, and mesocarp moisture of fruits sampled from olive trees (‘Frantoio’) subjected to FI or RF conditions.

Irrigation	Fruit FW (g)	Fruit DW (g)	Mesocarp FW (g)	Endocarp FW (g)	Mesocarp DW (g)	Endocarp DW (g)	Mesocarp to endocarp ratio (FW)	FW/DW fruit	Mesocarp moisture (%)
FI	2.60b	1.42b	1.78b	0.82b	0.90b	0.52	2.16b	1.84b	49.0b
RF	1.61a	1.10a	0.98a	0.63b	0.61b	0.49	1.59a	1.46b	37.6a
*LSD*	*0.28*	*0.13*	*0.18*	*0.14*	*0.08*	*0.08*	*0.36*	*0.27*	*7.73*

*Values are means of four trees per treatment (*n* = 4). Different letters indicate LSD between irrigation treatments ANOVA (*p* < 0.05).*

### Phenolic Composition in Fruit and VOO

**FIGURE 2:**
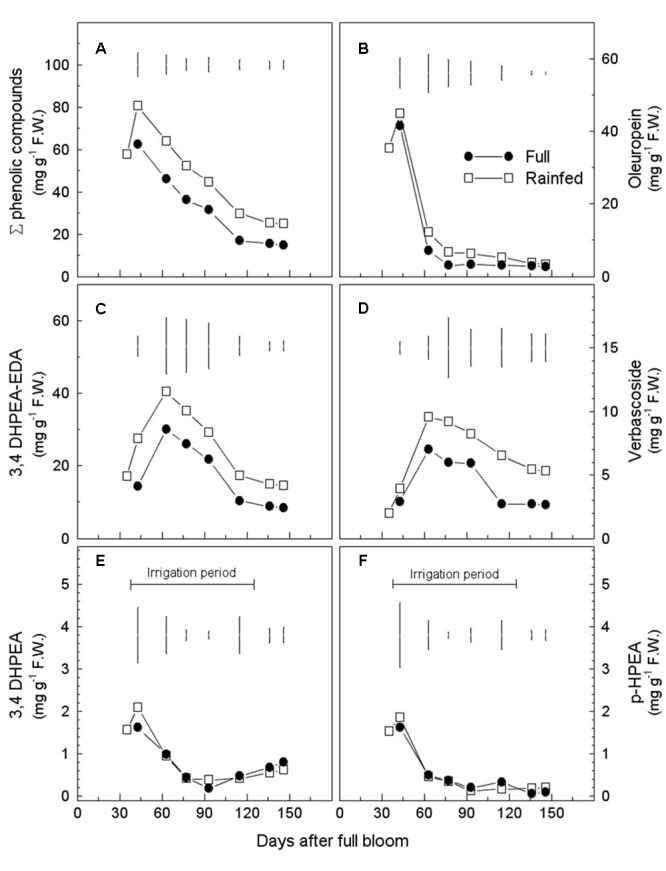
**Seasonal variations in phenolic compounds [mg (g FW)^-1^] in mesocarp of fruits from fully irrigated (FI) and rain-fed (RF) olive trees during fruit development.** Total phenol content is shown in **(A)**, oleuropein in **(B)**, 3,4-DHPEA-EDA in **(C)**, verbascoside in **(D)**, 3,4-DHPEA in **(E)** and *p*-HPEA in **(F)**. Values are means of three trees for each irrigation treatment. Vertical bars represent LSD between irrigation treatments ANOVA (*p* < 0.05).

Total phenols content (TPC), calculated as the sum of phenolic fractions on a mesocarp FW basis [mg (g FW)^-1^], showed a decreasing trend during fruit development, independently of the irrigation regime (**Figure 2A**). At maturity (146 DAFB), TPC was significantly higher in drupes of the RF trees than in those of the FI trees [24.7 vs. 14.8 mg (g FW)^-1^, respectively] (Supplementary Table S3). Differences in TPC between the two treatments were already evident at early stages of drupe development (43–65 DAFB) and were thereafter maintained without major changes until fruit maturity. Notably, the TPC content was higher in mature RF drupes on a DW basis (**Table 3**). As for individual fractions 3,4-DHPEA-EDA, verbascoside, and oleuropein contributed the most to the difference between mature fruits of FI and RF trees (**Table 3**). 3,4-DHPEA-EDA and verbascoside increased from 35 to 63 DAFB, but both tended to decrease later (**Figures 2C,D** and Supplementary Table S3). The oleuropein content was higher at the beginning of drupe development (from 35 to 43 DAFB) [60% of phenol fraction: 41.5 mg (g FW)^-1^] and after that strongly decreased, reaching a plateau at about 77 DAFB, under both water regimes (**Figure 2B** and Supplementary Table S3). The simple phenols 3,4-DHPEA and *p*-HPEA accounted for less than 3% of the phenol fraction analyzed (**Figures 2E,F** and Supplementary Table S3). Their content was higher at the early stage of fruit development and then declined sharply, apparently unaffected by water management. 3,4-DHPEA-EDA reached maximum content at 63 DAFB. Also, the lignans (+)-1-acetoxipinoresinol and (+)-1-pinoresinol, accounting for less than 1.5% of the phenol fraction, were unaffected by water status (Supplementary Table S4).

**Table 3:** Phenolic compounds in mesocarp of fruits [mg (g DW)^-1^] sampled at harvest (146 DAFB) and oils (mg kg^-1^) obtained from fruits sampled at the same data from olive trees subjected to FI or RF conditions.

Irrigation	*p-*HPEA	3,4-DHPEAz	Oleuropein	Verbascoside	3-4 DHPEA-EDA	Sum of phenolic fractions
FI	0.16	1.54	4.93	5.16	15.91a	28.47a
RF	0.34	0.99	5.40	8.57	23.56b	39.94b
*LSD*	*0.40*	*0.58*	*1.22*	*4.24*	*4.36*	*7.88*
Olive oil
FI	13.6	2.0	28.3a	35.7a	80.4ab	167.5a
RF	10.7	3.9	124.2	92.0b	133.3b	373.3b
*LSD*	*5.1*	*3.0*	*8.8*	*12.77*	*40.81*	*59.1*

*Values are means of three trees for each irrigation treatment. Different letters indicate LSD between irrigation treatments ANOVA (p < 0.05).*

The phenolic compounds concentration in VOO was also markedly affected by the soil water availability. The differences in TPC content were similarly significant in VOO, with contents in oils extracted from RF drupes more than twofold higher than those of oils extracted from FI drupes (**Table 3**). The 3,4-DHPEA-EA, p-HPEA-EDA, and the sum of phenolic fractions in VOO obtained from RF trees were significantly higher (438, 257, and 223%, respectively) than those from the FI trees.

### Changes in Enzyme Activities during Fruit Development

**FIGURE 3:**
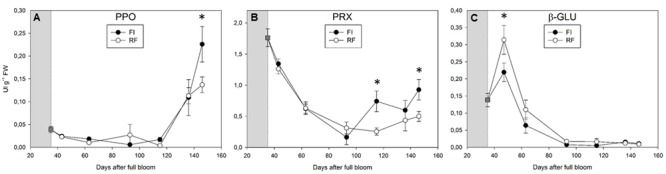
**Polyphenoloxidase (PPO) (A)**, peroxidase (PRX) **(B)**, and β-glucosidase (β-GLU) **(C)** activity detected in olive fruit of FI and RF trees. Dashed line indicates the beginning of treatment. Symbols and bars indicate the average and SD of four biological and two technical replications. Asterisks (^∗^) indicate a significant statistical difference between FI and RF treatments at *p* < 0.01 (Student’s *t*-test).

Polyphenoloxidase activity remained low throughout fruit development until the onset of fruit ripening (from 43 to 115 DAFB), without significant changes between FI and RF trees (**Figure 3A**). PPO activity increased during ripening (from 136 to 146 DAFB), with higher values in FI tress (**Figure 3A**). In both FI and RF trees, the levels of PRX activity progressively decreased during the first 93 days of ripening before increasing in the following 50 days. In fruits from FI trees, the PRX activity at day 146 was twofold higher than that in fruits from RF trees (**Figure 3B**). A peak of β-GLU activity was detected at about 50 DAFB in fruits from both FI and RF treatments, then this activity declined to become almost undetectable after the pit-hardening stage (**Figure 3C**).

### Identification of Transcripts Putatively Involved in Phenols Catabolism

A number of transcripts coding for PPO, PRX, and β-GLU enzymes and putatively expressed in fruit tissues have been identified.

Four full-length transcripts coding for *PPO* genes were arbitrarily named *OePPO1-like, 2-like, 3-like*, and *4-like*, after identification in a flower and fruit library (Supplementary Table S2). The isoforms were encoded by a single exon, as also reported in other plant species (Tran et al., 2012). Conceptual translation of the four putative *PPO* sequences allowed identification of the conserved tyrosinase domain (Supplementary Figure S2), composed of the copper-binding sub-domains CuA and CuB and characterized by six conserved histidine residues that bond the two copper ions of the active site (Tran et al., 2012). Moreover, two domains with unknown function, DWL (Pfam12142) and KFDW (Pfam12143), previously identified in *P. trichocarpa* PPOs, were also present (Supplementary Figure S2). A chloroplast localization signal was identified in all four olive *PPO* genes, as also predicted in other species (Supplementary Table S5). Phylogenetic analysis grouped OePPO1-like, 2-like, and 4-like in a clade comprising several PPO members of *Mimulus guttatus*, while OePPO3-like seemed to be phylogenetically distant (Supplementary Figure S3). Class III peroxidases are heme-containing glycoproteins encoded by a multigene family (Hiraga et al., 2001). At least 61 members of class III peroxidase gene family were identified in a draft assembly of *O. europaea* cv. ‘Leccino’ genome (data not shown), 5 of which resulted the most represented in RNA-seq libraries from olive fruit tissues. Active site residues of identified OePRXs contained the catalytic distal Arg38, His42 hydrogen-bonded to Asn70 (Supplementary Figure S4) (Smith and Veitch, 1998). Furthermore, we identified other conserved amino acid residues, such as Pro139, which putatively accepts a hydrogen bond from reducing substrates, and His170, which is coordinated to heme Fe^3+^ and hydrogen-bonded to Asp247. In OePRX42, the His70 was replaced by Ser70, and this substitution also occurred in other plant PRXs, such as *A. thaliana* PRX1 (Tognolli et al., 2002). Predicted side chain ligands to the distal and the proximal Ca^2+^ ions were also identified (Supplementary Figure S4). OePRXs contained eight conserved cysteine residues putatively involved in disulfide bridges and a buried salt bridge motif present in all class III PRXs (Welinder, 1992). Putative OePRXs were predicted as secretory proteins (Supplementary Table S6). Moreover, as in other species, OePRXs had several N-linked glycans in the sequence motif Asn-X-Ser/Thr (Veitch, 2004). Phylogenetic analysis confirmed the similarity of the identified olive PRXs with other class III PRXs (Supplementary Figure S5).

Amino acid alignments of the four identified members of the olive glycoside hydrolase (GH) family 1 highlight the presence of the two glutamate residues embedded in the highly conserved motifs TF/LNEP (acid/base catalyst) and I/VTENG (nucleophile), except for Oeβ-GLU46-like characterized by the motifs TVNEA/IHENG (Supplementary Figure S6). The analysis with TargetP v1.0 predicted that Oeβ-GLU11 and 46-like are localized in extra-cellular space, while the localization of both Oeβ-GLU12-like isoforms is uncertain (Supplementary Table S7). Phylogenetic analyses were performed to gain information about their putative substrates (Supplementary Figure S7). Oeβ-GLU12-like1 and Oeβ-GLU12-like2, both belonging to the GH1 subgroup 12, cluster in a well-differentiated clade composed of the recently characterized Oeβ-GLU, and RsSG and RsRG enzymes of *Rauvolfia serpentina*, involved in monoterpenoid indole alkaloids biosynthesis (Warzecha et al., 2000; Gerasimenko et al., 2002). It is unclear whether the two olive Oeβ-GLU12 isoforms are different alleles of the same isoform, since *Oeβ-GLU* is encoded by a single locus in olive cultivar ‘Koroneiki’ (Koudounas et al., 2015). Oeβ-GLU46 belongs to a large subgroup composed of glucosidases putatively involved in the phenylpropanoids pathway and lignin biosynthesis. Consistent with this hypothesis, Oeβ-GLU46 is predicted to be targeted to the extracellular space (Supplementary Table S7). Oeβ-GLU11 belongs to the AtGLU11 subgroup, the members of which seem to work on different substrates, such as hydroxyisourate for the biosynthesis of the allantoin precursor in *Glycine max* (Raychaudhuri and Tipton, 2002).

### Relative Gene Expression

**FIGURE 4:**
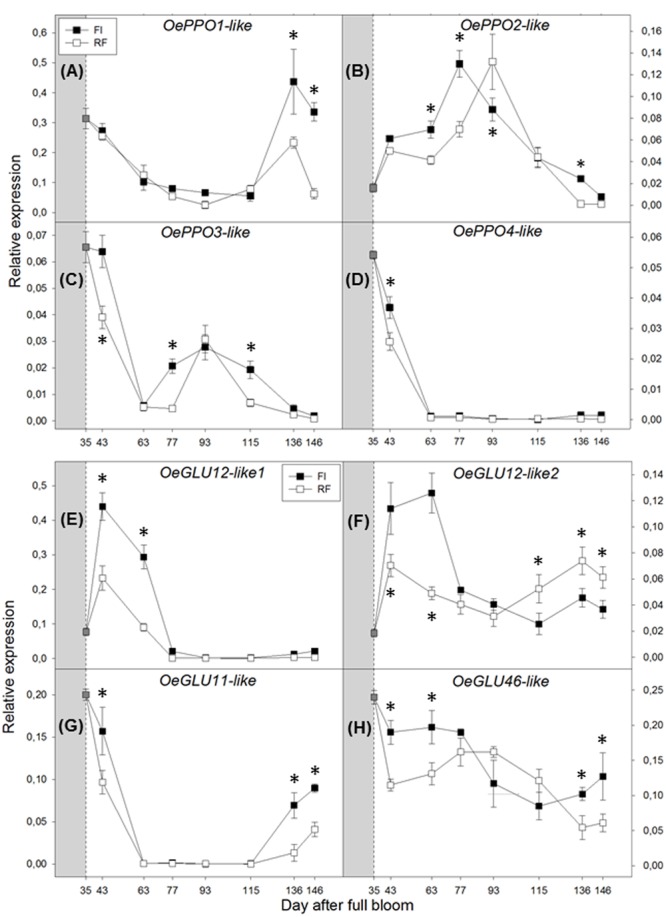
**Real-time quantitative PCR analysis of *OePPO1-like* (A)**, *OePPO2-like* **(B)**, *OePPO3-like* **(C)**, *OePPO4-like* **(D)**, *OeGLU12-like1* **(E)**, *OeGLU12-like-2* **(F)**, *OeGLU46-like* **(G)**, and *OeGLU11-like* **(H)**, in FI and RF trees. Dashed line indicates the beginning of treatment. Symbols indicate the average of four biological replications, and bars show ± SD. Asterisks (^∗^) indicate a significant statistical difference between FI and RF treatments at *p* < 0.01 (Student’s *t*-test).

The expression of *OePPO* genes was differently modulated along the stages of drupe development and appeared to be affected by plant water status. *OePPO1* expression was high during young fruit development, decreased after 43 DAFB, and strongly increased again at the two late sampling dates (136 and 146 DAFB) (**Figure 4A**). This expression pattern may suggest that OePPO1 is the main enzymatic isoform active during ripening. Moreover, *OePPO1* was expressed twofold more in drupes of FI trees. By contrast, *OePPO2* showed the highest peak of transcripts during the pit-hardening phase (from 77 to 93 DAFB), with the maximum expression value in FI trees reached earlier than that in RF trees (**Figure 4B**). *OePPO3* and *OePPO4* were expressed at lower levels, although the former was up-regulated from 77 to 115 DAFB in fruits of FI trees and only at 93 DAFB in fruits of RF trees, whereas the latter gene was downregulated from 63 DAFB until the harvest of fruits (**Figures 4C,D**).

Among the *Oeβ-GLU* genes analyzed, *Oeβ-GLU12.1* transcripts showed the highest magnitude and were essentially expressed early in drupe development (from 43 to 63 DAFB) (**Figure 4E**). The other isoform, *Oeβ-GLU12.2*, showed two peaks, one at the beginning of drupe growth (35 DAFB) and the other near ripening (146 DAFB) (**Figure 4F**). Both *Oeβ-GLU12* genes showed higher transcript levels in FI trees. *Oeβ-GLU11* showed higher expression in young fruit that then increased again at ripening, more in FI drupes (**Figure 4G**). *Oeβ-GLU46* expression did not seem to be affected by either fruit development or water availability (**Figure 4H**).

**FIGURE 5:**
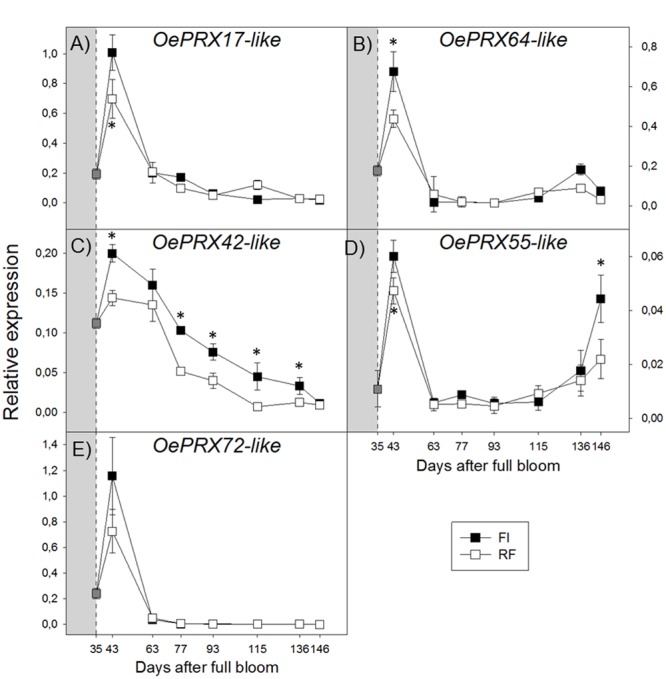
**Real-time quantitative PCR analysis of *OePRX17-like* (A)**, *OePRX64-like* **(B)**, *OePRX42-like* **(C)**, *OePRX55-like* **(D)**, and *OePRX72-like* **(E)**, in FI and RF trees. Dashed line indicates the beginning of treatment. Symbols indicate the average of four biological replications, and bars show ± SD. Asterisks (^∗^) indicate a significant statistical difference between FI and RF treatments at *p* < 0.01 (Student’s *t*-test).

Transcript levels of all *OePRX* genes were higher during the earlier stages of fruit development and then sharply decreased. At ripening, *OePRX17* and *OePRX72* were barely detectable (**Figures 5A,E**), while *OePRX64* and *OePRX55* increased again, more in FI fruit (**Figures 5B,D**). *OePRX42* showed an expression pattern similar to that of *OePRX17*, although the reduction of transcript levels during fruit development was less drastic (**Figure 5C**).

## Discussion

We confirmed the general decrease in phenolics content during olive fruit development reported in previous work (Alagna et al., 2012) but, interestingly, each metabolite showed a specific pattern (**Figure 2**). An inverse relationship between oleuropein content and its derivative 3,4-DHPEA-EDA appeared evident during early stages of fruit development. The up-regulation of both identified *Oeβ-GLU12-like* isoforms, showing a high similarity with *Oeβ-GLU* (involved in oleuropein deglycosylation), and the peak of β-GLU activity further supported their predominant role in the oleuropein catabolic processes occurring during early stages of fruit development and contributing to the composition of olive phenolics (**Figures 3C, 4E,F**). These results agree with those reported by Gutierrez-Rosales et al. (2010), who hypothesized that 3,4-DHPEA-EDA was formed via oleuropein by β-GLU activity. Transcription levels of *OeGLU12*-*like1* and *like2* were higher in FI trees, unlike enzyme activity and 3,4-DHPEA-EDA content, which were both higher in RF trees. Although a positive effect of water deficit on GLU activity could be hypothesized, the identification of putative esterase enzyme(s) regulating 3,4-DHPEA-EDA biosynthesis will help to better elucidate this pathway and the effect of water availability.

The expression of olive *PPO* genes was affected by olive fruit developmental stages as well as plant water status (**Figure 4**). *OePPO1* seems to be the main isoform during early fruit development and maturation (after 115 DAFB), whereas *OePPO2* was mainly expressed during the pit-hardening period (from 77 to 93 DAFB). A relationship between the expression of olive *PPO* genes and PPO activity appeared evident during fruit ripening (**Figures 3A, 4**), and a close relationship between drupe PPO activity and fruit developmental stage has already been observed in cultivar ‘Zard,’ accompanied by differential expression of isoenzymes (Ebrahimzadeh et al., 2003). Significant changes in kinetic behavior of PPO were observed also during fruit maturation in cultivar ‘Picual,’ and it was suggested that the assayed activity may be the result of the expression of different genes (Ortega-García et al., 2008). Transcript levels of *OePPO1* and PPO activity were higher in irrigated trees during advanced stages of fruit maturation, but gene expression and activity were not correlated with phenolic amount or specific metabolites, which remained stable during ripening. This agrees with the findings of Ortega-García et al. (2008), who showed that the increase in PPO activity during fruit ripening of cultivar ‘Picual’ did not parallel the variation in oleuropein concentration. Therefore, in intact olive drupes, PPO activity apparently plays a minor role in secoiridoid metabolism.

Peroxidases (Class III) oxidize phenolics as preferential substrates at the expense of peroxides. The highest levels of *OePRX* transcripts were detected from 35 to 43 DAFB. *OePRX* transcripts accumulation decreased sharply after the first stage of growth (Supplementary Figure S2). However, levels of *OePRX64* and *OePRX55* increased again in ripe fruits. In most cases, the expression levels were higher in FI trees than in RF trees, and we generally found a good correlation between *PRX* transcript accumulation and PRX activity: the highest enzyme activity was detected early during fruit development (35 DAFB), subsequently it decreased and then increased again at maturation (**Figure 3B**). High levels of PRX activity have been found in olive seed, and the effect of this enzymatic activity on the VOO phenolic profile has been proved (Luaces et al., 2007). A PRX enzyme that binds specifically to pectic polysaccharides has been purified from black ripened olives (‘Douro’) (Saraiva et al., 2007), and more recently Tzika et al. (2009) have reported the partial purification of a PRX enzyme from ‘Koroneiki’ olive fruits that seems to be active toward some olive fruit phenols but inactive toward oleuropein. Contribution of PRX to the oxidation of phenols is generally limited by the availability of H_2_O_2_, which usually increases under stress conditions or tissue damage (Sharma et al., 2012). The expression pattern and activity of several PRX isoforms was well correlated with phenol content in immature fruits but seemed to be unaffected by water availability, since the levels of PRX activity were similar between FI and RF treatments. Considering that a direct involvement of PRXs in oleuropein oxidation has yet to be demonstrated, such activity does not appear to play a pivotal role in explaining the different phenolics contents found in immature drupes.

Water availability is one of the major factors affecting olive yield and quality of both table olives and olive oils, particularly in areas where long periods of summer drought occurs during fruit growth and development. It has been shown that water availability affects the content of olive phenolics in fruit and oil and that the increase in irrigation volumes is negatively correlated with secoiridoids content (Patumi et al., 1999, 2002; Tovar et al., 2001, 2002; Gümez-Rico et al., 2006; Servili et al., 2007b; Caruso et al., 2014). In our study, fruits and oils from RF trees had consistently higher phenolic contents than those from FI trees. The ratio of phenolic content between FI and RF trees was 71 and 45% in the mesocarp (DW basis) and VOO, respectively, indicating that the RF conditions probably increased the fraction of phenols transferring into VOO during extraction. Nonetheless, there are many intriguing issues that remain to be clarified. For example, differences in fruit phenolic contents arose soon after the beginning of irrigation (35–43 DAFB), when drupe size was not significantly different between FI and RF trees, and thereafter remained almost unchanged until maturity, despite the progressive decrease in PLWP and fruit enlargement. Therefore, we hypothesize that phenolic catabolism is particularly sensitive to water availability during early stages of drupe development, affecting oleuropein catabolism through the regulation of glucosidase activity. As already reported in other species, the timing of water deficit, and its intensity and duration, is crucial for assimilate partitioning between primary and secondary pathways (Ni et al., 2009; Ripoll et al., 2014). In olive fruits, moderate water deficit starting 63 DAFB had no effects on cell division, but reduced mesocarp cell expansion, FW, and, only slightly and later in the mature stage of development, DW, suggesting that different cellular processes are involved depending on the stage of fruit development (Gucci et al., 2009, 2011). Analogously, we hypothesize that a cross-talk is active between fruit development and cell water status for regulating the expression of genes and activity of enzymes playing a role in phenolic catabolism, but further studies are needed to clarify this point.

## Conclusion

We have provided integrated evidence concerning the regulation and role of GLU, PRX, and PPO in the accumulation of phenolic compounds in olive fruits, suggesting a key role for β-GLU in oleuropein catabolism. Moreover, we provide the first detailed study on the effect of water availability on phenolics metabolism during fruit growth and ripening, suggesting that fruit response may vary depending on the developmental stage and the timing of water deficit. Knowledge of the genetic and physiological mechanisms underlying plant responses to the frequent succession of severe drought periods becomes a crucial factor, especially in optimizing irrigation management to achieve a balanced trade-off between olive oil yield and quality, and the saving of water resources.

## Author Contributions

RG, MS, MR, EP, MC, and RM developed the concept of the paper, wrote the paper, GC and CG performed eco-physiological analysis, collected and analysed meteorological data, MC and EF carried out qRT-PCR analyses and bioinformatic analyses, SU performed HPLC-polyphenolic analysis and quantification, and EF, EP, and MR performed enzymatic activities. All authors discussed and commented the manuscript.

## Conflict of Interest Statement

The authors declare that the research was conducted in the absence of any commercial or financial relationships that could be construed as a potential conflict of interest.

## Footnotes

1 http://biolign.software.informer.com/4.0/

2 http://140.164.45.140/oleaestdb/search.php

3 http://www.Primer3.com

4 http://www.ebi.ac.uk/Tools/msa/clustalw2/

5 https://phytozome.jgi.doe.gov/pz/portal.html

6 http://www.cbs.dtu.dk/services
