# Supplementary material for: The Role of Polyphenoloxidase, Peroxidase, and β-Glucosidase in Phenolics Accumulation in Olea europaea L. Fruits under Different Water Regimes
Source: Front Plant Sci. 2017 May 9;8:717. doi: 10.3389/fpls.2017.00717 (PMC5422556; doi:10.3389/fpls.2017.00717)
Supplement: Supplementary file 1 [file Data_Sheet_1.PDF]

Supplementary Material

The role of polyphenoloxidase, peroxidase and β-glucosidase in phenolics accumulation in *Olea europaea* L. fruits under different water regimes

Marco Cirilli[1,±], Giovanni Caruso[2], Clizia Gennai[2], Stefania Urbani[3], Eleonora Frioni[1], Maurizio Ruzzi[4], Maurizio Servili[3], Riccardo Gucci[2], Elia Poerio[4], Rosario Muleo[1*]

[1]Dipartimento di Scienze Agrarie e Forestali, Laboratorio di Ecofisiologia Molecolare delle Piante Arboree, Università degli Studi delle Tuscia, Via S. C. De Lellis snc, 01100, Viterbo, Italy

[2]Dipartimento di Scienze Agrarie, Alimentari e Agro-ambientali, Università di Pisa, Via del Borghetto 80, 56124, Pisa, Italy

[3]Dipartimento di Scienze Agrarie, Alimentari ed Ambientali, Università degli studi di Perugia, Borgo XX Giugno, 74, 06121, Perugia, Italy

[4]Dipartimento per la Innovazione nei Sistemi Biologici, Agro-alimentari e Forestali, Via Camillo De Lellis snc, 01100, Viterbo, Italy

[±]Present address: Department of Agricultural and Environmental Sciences, University of Milan, Milan, Italy

Supplementary Table S.1. Primer sequences of amplified target genes, PCR efficiency calculated from standard curve slope of target genes, and product size (bp) of amplified fragments**.**

Gene target	Primer Forward (5'-3')	Primer Reverse (5'-3')	PCR efficiency	Amplicon size
OeEF-1α	ACCACTGGTGGTTTTGAAGC	GAAACCAGAGATGGGGACAA	1.99	234
*OePPO*1-like	GCCAGGGTGATAAAGACTACTTCC	GCAGTTCATCAGCTTTACCCTTC	1.98	121
*OePPO*2-like	GGTTGAACGCTAGTTTCGTGTTCT	CTTGATGTCTGCTGTTGATGGAG	1.97	200
*OePPO*3-like	GATTGTTAGGGTCCACTGAGGTTC	CCTATTAGGGACGGTGATGATGTC	2.00	112
*OePPO*4-like	CTCAATGAAATACTAGACTTGCCC	TCAAGACATACAGACAGACACTAC	1.99	165
*OePRX*17-like	TTCTGACGATATAATGCCAAGTCC	GGTCTTCCTGTACCTGATTGATTA	1.99	177
*OePRX*29-like	CCTACTACTCCAGCAGCTATTTTA	TCGTGTTTATTGTCTCCCTCTTAC	1.99	160
*OePRX*42-like	GATAACAAGGGACTGTTGATTGTG	TTGAAGAAATAGTCCTGGCTCTTG	1.98	178
*OePRX*55-like	GCTTCAGTTCTTATAGCTTCACCA	GGTTAAAACGTCTGCACATGATAC	1.98	156

*OePRX*72-like	CAACATTCCTGCTCCTAATAACAC	GGTTTAGCATTTCCTGATTGGTTG	1.98	168
*Oeβ-GLU*12.1	GATTCACTATCACCAAGAACATC	TACCAAATCTGACACTATATCCC	1.99	149
*Oeβ-GLU*12.2	GATACAGTGTTAGATTTGGCCTT	CTATACCCTTCTTCATTGTCTTG	1.99	154
*Oeβ-GLU*46	TTTGCCATTCAGGCTTATCGTTT	CTCCTTGTTGCTTCTTGTATTTAG	1.98	178
*Oeβ-GLU*11	CTATGTGGTATGAACCCTACACC	GTCAACTGAACCCTTAATCTGCTC	1.97	194

Supplementary Table S.2. Gene models available from *Phytozome* v11.0 used for phylogenetic analysis. Accession number, presence of intron(s), amino acidic size of proteins (AA) and transcript name pertaining to this paper are reported.

PPO gene family models

Genome	Accession number	Intron(s)	AA	Transcript name
Popolus trichocarpa	POPTR_0011s10950	0	563	PtrPPO1
	POPTR_0001s39630	0	581	PtrPPO2
	POPTR_0011s04710	0	590	PtrPPO3
	POPTR_0001s39920	0	581	PtrPPO5
	POPTR_0001s39950	0	606	PtrPPO11
	POPTR_0001s39940	1	553	PtrPPO12
	POPTR_0001s39660	0	581	PtrPPO14
	POPTR_0001s39680	0	581	PtrPPO15
Vitis vinifera	GSVIVT00011782001	0	607	VviPPO1
	GSVIVT00011776001	1	584	VviPPO2
	GSVIVT00011780001	0	607	VviPPO3
	GSVIVT00036366001	0	538	VviPPO4
Prunus persica	ppa003257m	0	589	PpePPO1
	ppa003608m	1	562	PpePPO2
	ppa019877m	0	619	PpePPO3
	ppa003190m	0	594	PpePPO4
Malus domestica	MDP0000699845	1	569	MdPPO1
	MDP0000500159	1	549	MdPPO2
	MDP0000234782	0	610	MdPPO3
	MDP0000744636	1	549	MdPPO4
	MDP0000298729	2	424	MdPPO5
Zea mays	GRMZM2G108103_T01	1	569	ZmaPPO1
	GRMZM2G121605_T01	2	629	ZmaPPO2
	AC233851.1_FGT017	1	597	ZmaPPO3
	GRMZM2G319062_T01	1	645	ZmaPPO4
	AC209206.3_FGT014	2	546	ZmaPPO6
Aquilegia coerulea	Aquca_003_00253.1	1	593	AcoPPO1
	Aquca_014_00431.1	2	587	AcoPPO2

Aquca_028_00118.1	2	593	AcoPPO4
Aquca_054_00045.1	0	587	AcoPPO5
Aquca_054_00047.1	2	586	AcoPPO6

PRX gene family model

Genome	Accession number[a]	Intron(s)	AA	Transcript name
Arabidopsis thaliana	At3g49110	3	354	AtPCa
	At3g49120	3	353	AtPCb*
	At1g05260	3	326	AtPRC
	At5g06720	3	335	AtPA2
	At2g38380	3	349	AtPEa
	At5g19890	3	328	AtPN
	At4g21960	3	330	AtP1*
	At2g37130	4	327	AtP2
	At5g64100	2	331	AtP3
	At1g71695	2	258	AtP4
	At1g49570	3	350	AtP5
	At5g66390	3	336	AtP6*
	At3g50990	3	336	
	At3g21770	3	329	AtP7
	At4g30170	3	325	AtP8
	At4g37520	3	329	AtP9
	At5g67400	3	329	AtP10
	At1g05240	3	325	AtP11
	At1g05250	3	325	
	At3g01190	3	321	AtP12
	At5g17820	2	313	AtP13
	At5g22410	3	331	AtP14

At5g64120	2	328	AtP15
At3g32980	3	352	AtP16*
At5g42180	3	317	AtP17
At1g44970	3	346	AtP18
At4g11290	3	326	AtP19
At5g14130	2	330	AtP20*
At3g49960	3	329	AtP21
At2g18980	3	323	AtP22
At1g68850	3	336	AtP23
At4g16270	3	362	
At5g39580	2	319	AtP24
At2g41480	3	328	
At1g77100	3	336	
At4g25980	3	326	
At2g22420	2	329	AtP25*
At5g40150	0	328	AtP26
At5g51890	2	322	AtP27
At2g35380	2	336	AtP28
At5g06730	3	358	AtP29*
At1g30870	1	349	AtP30
At2g24800	3	329	
At4g36430	3	331	AtP31
At2g18140	3	337	
At4g33420	3	314	AtP32*
At4g33870	3	404	
At5g15180	3	329	AtP33
At2g38390	3	349	AtP34
At4g26010	1	310	AtP35
At2g18150	3	338	AtP36
At4g37530	3	329	AtP37
At4g08770	3	346	AtP38

	At4g08780	3	346	
	At3g03670	2	321	AtP39
	At3g17070	2	339	AtP40*
	At3g28200	0	316	AtP41
	At5g19880	3	329	AtP42
	At5g47000	0	334	AtP43
	At4g17690	0	326	
	At1g24110	0	326	
	At5g58390	2	316	AtP44
	At5g58400	2	325	
	At5g64110	2	330	AtP45
	At1g14540	3	315	AtP46
	At1g14550	3	321	
	At2g39040	3	350	AtP47
	At4g31760	4	354	AtP48
	At5g05340	3	324	AtP49
	At2g43480	3	335	AtP50
	At5g24070	3	340	
	At2g34060	2	346	AtP51
Vitis vinifera	GSVIVT01003417001	3	361	VviPOX6
	GSVIVT01025650001	4	328	VviPOX38
	GSVIVT01034984001	2	325	VviPOX82
	GSVIVT01012727001	4	178	VviPOX8
	gi|147853497:7514-9230		318	VviPOX65*
	GSVIVT01018865001	5	235	VviPOX16
Prunus persica	ppa008698m	2	322	PpePOX25
	ppa008728m	2	321	PpePOX27
	ppa018671m	2	317	PpePOX52
	ppa008634m	2	324	PpePOX21*
	ppa008503m	3	329	PpePOX12
	ppa008569m	3	326	PpePOX17
	ppa008349m	3	336	PpePOX6

	29772.m000315	3	268	RCPOX17*
Ricinus communis	30190.m010916	2	330	RcPOX55
	29634.m002067	3	331	RcPOX72
	Potri.007G096200	2	330	PtrPOX35
	Potri.005G072800	2	330	PtrPOX59
	Potri.004G015300	4	331	PtrPOX2
Popolus trichocarpa	Potri.017G064100	2	323	PtrPOX82
	Potri.001G329200	2	314	PtrPOX21
	Potri.005G118700	3	333	PtrPOX45
	Potri.007G019300	3	333	PtrPOX49

Gene structures and amino acid sequences of putative *OePPO*, *OePRX* and *Oeβ-GLU* genes

**Gene**	**Exon**	**AA**
OePPO1-like	1	587
OePPO2-like	1	573
OePPO3-like	1	584
OePPO4-like	1	582
OePRX17-like	3	339
OePRX64-like	3	314
OePRX42-like	3	329
OePRX55-like	3	323
OePRX72-like	3	333
Oe-GLU12-like2	11	552
Oe-β-GLU46-like	8	517
Oeβ-GLU12-like1	11	550
Oeβ-GLU11-like	7	511

Supplementary Table S.3. Accumulation of phenolic compounds (mg/g FW fruit tissue) during olive drupe development and ripening in full irrigation (FI) and rain-fed (RF) tress of cv 'Frantoio'. Fresh weight; FW.

DAFB	3,4-DHPEA*z*		*p*HPEA		Verbascoside		3,4-DHPEA-EDA		Oleuropein		Sum of phenolic fractions	
	FI	RF	FI	RF	FI	RF	FI	RF	FI	RF	FI	RF
35	1.57±0.05		1.5±0.26		1.97±0.61		17.05±2.63		35.25±5.73		57.66±4.15	
43	1.62±0.80	2.09±0.10	1.6±0.94	1.9±0.11	2.88±0.12	3.90±0.5	14.39±0.3	27.40±2.5	41.52±1.0	44.96±0.5	62.26±3.9	80.46±5.6
63	0.99±0.43	0.95±0.33	0.5±0.03	0.4±0.24	6.99±0.61	9.54±0.9	30.3±3.66	43.83±3.6	7.19±1.72	8.79±0.87	46.11±3.3	63.95±4.7
77	0.44±0.08	0.43±0.13	0.4±0.08	0.4±0.04	5.99±0.20	9.20±2.9	26.06±3.9	36.68±5.4	3.13±1.67	5.04±1.45	36.37±2.8	52.14±2.1
93	0.19±0.05	0.38±0.10	0.2±0.15	0.1±0.14	5.91±0.27	8.22±1.8	21.71±1.4	30.93±5.0	3.37±1.74	4.50±1.00	31.62±2.8	44.42±3.2
115	0.47±0.45	0.41±0.30	0.3±0.42	0.2±0.13	2.68±1.30	6.50±1.2	10.37±2.7	17.22±1.9	3.02±1.22	5.25±2.28	17.11±2.6	29.80±1.2
136	0.68±0.11	0.54±0.19	0.1±0.02	0.2±0.14	2.71±0.90	5.42±1.0	8.75±0.99	14.99±1.2	2.84±0.28	3.64±0.53	15.43±1.4	25.37±1.7
146	0.80±0.07	0.61±0.21	0.1±0.02	0.2±0.15	2.64±0.98	5.31±0.9	8.30±0.90	14.61±1.5	2.57±0.20	3.35±0.30	14.79±1.4	24.75±2.1

Supplementary Table S.4. Lignans accumulation (mg/g FW) during olive drupe development and ripening in full irrigation (FI) and rainfed (RF) plants of cv 'Frantoio'. Fresh weight; FW.

DAFB	Acetoxypinoresinol		Pinoresinol	
	FI	RF	FI	RF
35	0.09±0.02		0.19±0.01	
43	0.09±0.01	0.09±0.01	0.13±0.08	0.16±0.08
63	0.19±0.09	0.20±0.12	0.22±0.11	0.20±0.09
77	0.19±0.09	0.18±0.09	0.20±0.08	0.24±0.11
93	0.09±0.05	0.06±0.01	0.15±0.09	0.19±0.10
115	0.08±0.05	0.09±0.05	0.14±0.08	0.15±0.09
136	0.21±0.08	0.29±0.14	0.18±0.01	0.31±0.25
146	0.20±0.08	0.29±0.10	0.20±0.03	0.39±0.30

Supplementary Table S.5. TargetP v1.0 prediction results of olive PPOs intracellular localization.

Name	Len	cTP	mTP	SP	Other	Loc	RC	TPlen
OePPO1	114	0.917	0.047	0.039	0.047	C	1	50
OePPO2	114	0.823	0.095	0.026	0.117	C	2	38
OePPO3	114	0.632	0.158	0.232	0.031	C	5	51
OePPO4	114	0.948	0.377	0.009	0.012	C	3	46

Supplementary Table S.6. TargetP v1.0 prediction results of olive PRXs intracellular localization.

Name	Len	cTP	mTP	SP	other	Loc	RC	TPlen
OePRX17	329	0.138	0.034	0.700	0.063	S	3	20
OePRX64	320	0.010	0.032	0.894	0.054	S	1	19
OePRX42	328	0.002	0.044	0.967	0.112	S	1	22
OePRX55	327	0.002	0.085	0.931	0.052	S	1	23
OePRX72	333	0.003	0.121	0.781	0.041	S	2	23

Supplementary Table S.7. TargetP v1.0 prediction results of olive *β*-GLUs intracellular localization.

Name	Len	cTP	mTP	SP	other	Loc	RC	TPlen
Oeβ-GLU12.1	495	0.728	0.054	0.117	0.632	*	5	-
Oeβ-GLU12.2	552	0.210	0.093	0.154	0.854	-	2	-
Oeβ-GLU46	517	0.027	0.018	0.965	0.060	S	1	24
Oeβ-GLU11	511	0.013	0.020	0.978	0.129	S	1	23

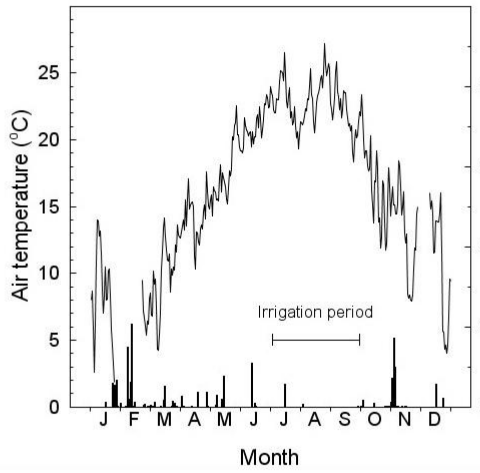

Supplementary Figure S.1: Daily values of mean air temperature (line) and total precipitation (histograms) at the experimental site in 2011. The irrigation period is also reported.

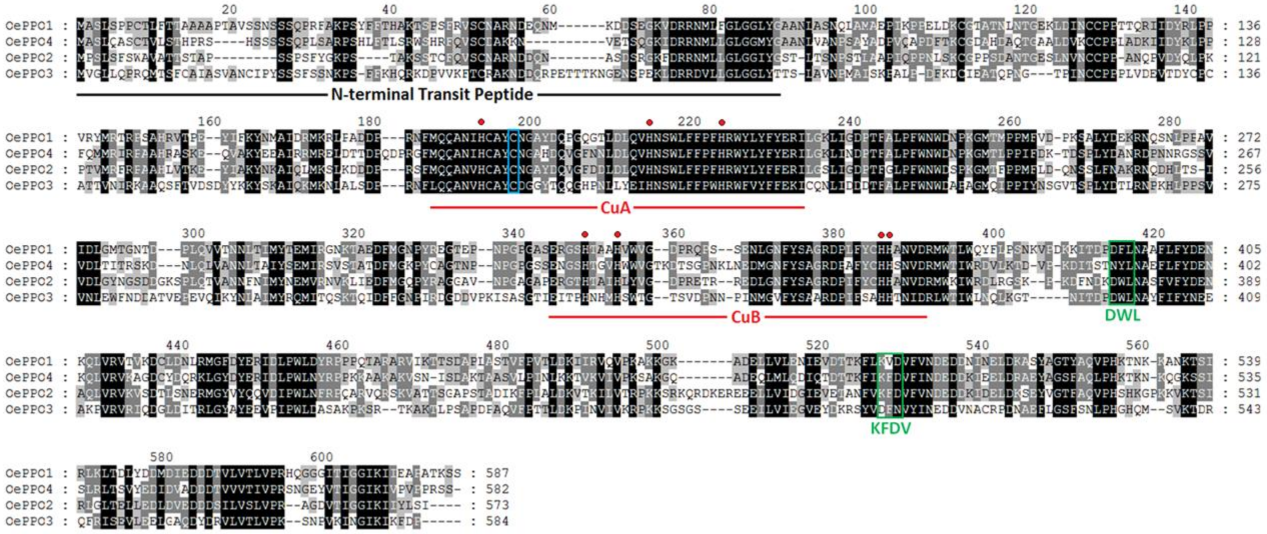

Supplementary Figure S.2. Sequence alignment of predicted olive PPO proteins. The conserved histidine residues of CuA and CuB binding domain are indicated by red circle; Cys residue of HXXXC sequence motif involved in thioether bound by blue colored box and DWL and KFDV domains by green colored box.

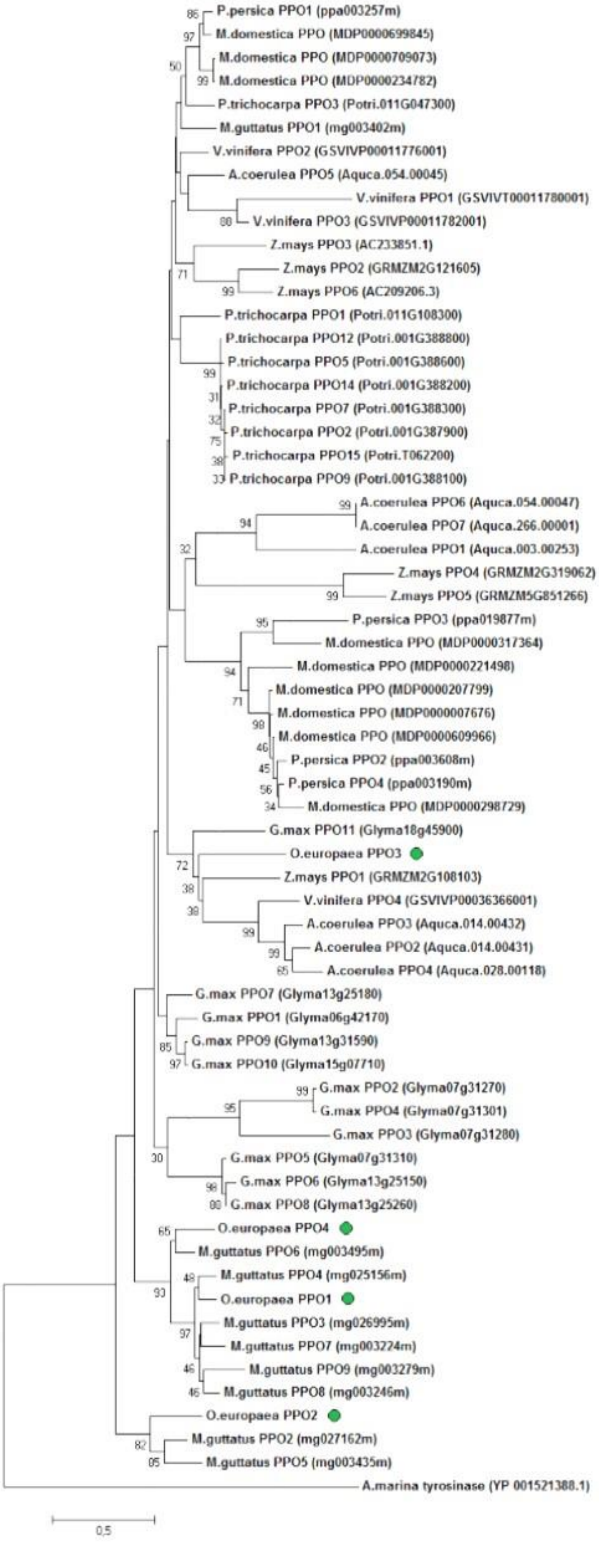
P.persica PPO1 (ppa003257m)
M.domestica PPO (MDP0000699845)
M.domestica PPO (MDP0000709073)
M.domestica PPO (MDP0000234782)
P.trichocarpa PPO3 (Potri.011G047300)
M.guttatus PPO1 (mg003402m)
V.vinifera PPO2 (GSVIVP00011776001)
A.coerulea PPO5 (Aquca.054.00045)
V.vinifera PPO1 (GSVIVT00011780001)
V.vinifera PPO3 (GSVIVP00011782001)
Z.mays PPO3 (AC233851.1)
Z.mays PPO2 (GRMZM2G121605)
Z.mays PPO6 (AC209206.3)
P.trichocarpa PPO1 (Potri.011G108300)
P.trichocarpa PPO12 (Potri.001G388800)
P.trichocarpa PPO5 (Potri.001G388600)
P.trichocarpa PPO14 (Potri.001G388200)
P.trichocarpa PPO7 (Potri.001G388300)
P.trichocarpa PPO2 (Potri.001G387900)
P.trichocarpa PPO15 (Potri.T062200)
P.trichocarpa PPO9 (Potri.001G388100)
A.coerulea PPO6 (Aquca.054.00047)
A.coerulea PPO7 (Aquca.266.00001)
A.coerulea PPO1 (Aquca.003.00253)
Z.mays PPO4 (GRMZM2G319062)
Z.mays PPO5 (GRMZM5G851266)
P.persica PPO3 (ppa019877m)
M.domestica PPO (MDP0000317364)
M.domestica PPO (MDP0000221498)
M.domestica PPO (MDP0000207799)
M.domestica PPO (MDP0000007676)
M.domestica PPO (MDP0000609966)
P.persica PPO2 (ppa003608m)
P.persica PPO4 (ppa003190m)
M.domestica PPO (MDP0000298729)
G.max PPO11 (Glyma18g45900)
O.europaea PPO3
Z.mays PPO1 (GRMZM2G108103)
V.vinifera PPO4 (GSVIVP00036366001)
A.coerulea PPO3 (Aquca.014.00432)
A.coerulea PPO2 (Aquca.014.00431)
A.coerulea PPO4 (Aquca.028.00118)
G.max PPO7 (Glyma13g25180)
G.max PPO1 (Glyma06g42170)
G.max PPO9 (Glyma13g31590)
G.max PPO10 (Glyma15g07710)
G.max PPO2 (Glyma07g31270)
G.max PPO4 (Glyma07g31301)
G.max PPO3 (Glyma07g31280)
G.max PPO5 (Glyma07g31310)
G.max PPO6 (Glyma13g25150)
G.max PPO8 (Glyma13g25260)
O.europaea PPO4
M.guttatus PPO6 (mg003495m)
M.guttatus PPO4 (mg025156m)
O.europaea PPO1
M.guttatus PPO3 (mg026995m)
M.guttatus PPO7 (mg003224m)
M.guttatus PPO9 (mg003279m)
M.guttatus PPO8 (mg003246m)
O.europaea PPO2
M.guttatus PPO2 (mg027162m)
M.guttatus PPO5 (mg003435m)
A.marina tyrosinase (YP 001521388.1)
0.5

Supplementary Figure S.3. Neighbour-joining tree generated using amino acid sequences for putative olive polyphenoloxidase proteins and PPOs from others species. Bootstrap replicates were used to determine the level of support at each node. Accession number are indicated in brackets**.**

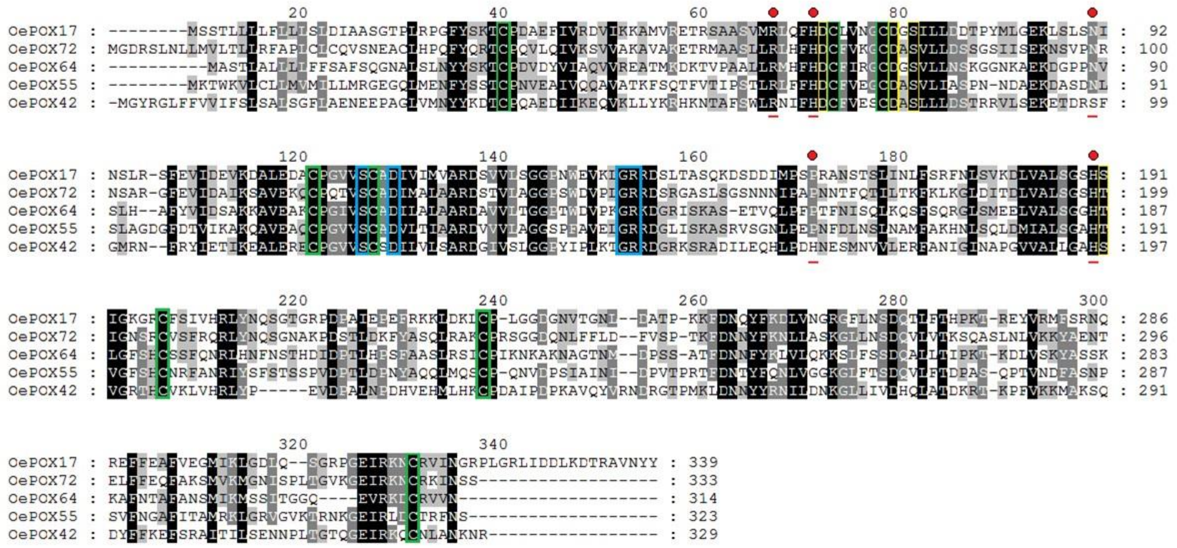

Supplementary **Figure S.4.** Sequence alignment of predicted olive PRXs proteins. Active site residues are indicated by red circle; side chain ligands to Ca^{2+} ions, cysteine residues and ion-pair motif are highlighted by yellow, green and blue boxes, respectively.

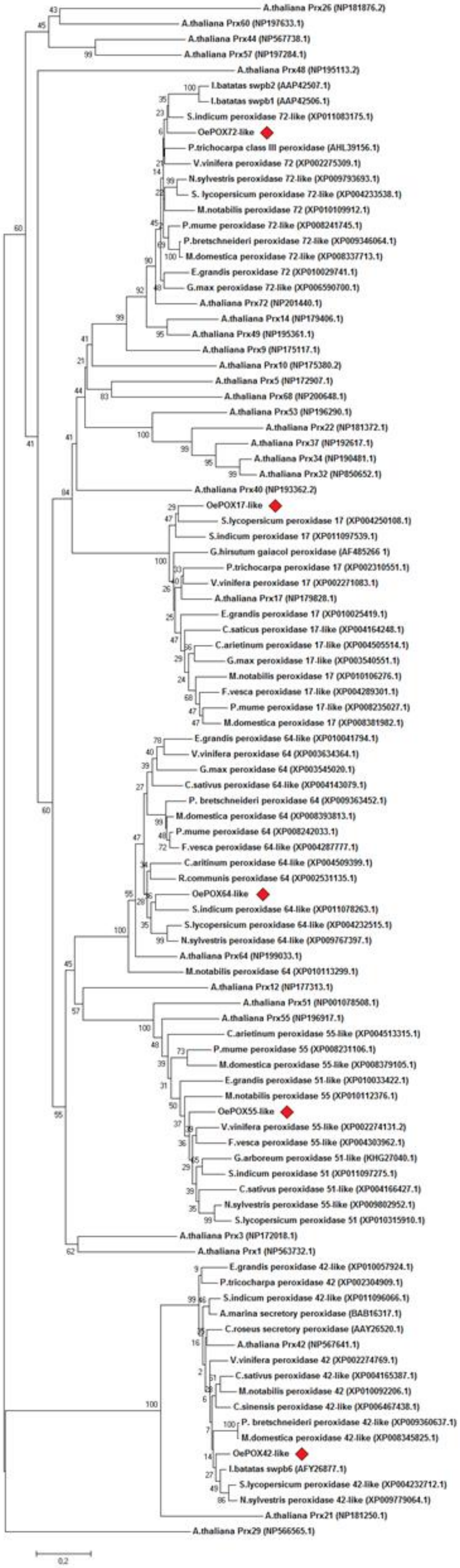
A.thaliana Prx26 (NP181876.2)
A.thaliana Prx60 (NP197633.1)
A.thaliana Prx44 (NP567738.1)
A.thaliana Prx57 (NP197284.1)
A.thaliana Prx48 (NP195113.2)
I.batatas swpb2 (AAP42507.1)
I.batatas swpb1 (AAP42506.1)
S.indicum peroxidase 72-like (XP011083175.1)
OePOX72-like
P.trichocarpa class III peroxidase (AHL39156.1)
V.vinifera peroxidase 72 (XP002275309.1)
N.sylvestris peroxidase 72-like (XP009793693.1)
S. lycopersicum peroxidase 72-like (XP004233538.1)
M.notabilis peroxidase 72 (XP010109912.1)
P.mume peroxidase 72-like (XP008241745.1)
P.bretschneideri peroxidase 72-like (XP009346064.1)
M.domestica peroxidase 72-like (XP008337713.1)
E.grandis peroxidase 72 (XP010029741.1)
G.max peroxidase 72-like (XP006590700.1)
A.thaliana Prx72 (NP201440.1)
A.thaliana Prx14 (NP179406.1)
A.thaliana Prx49 (NP195361.1)
A.thaliana Prx9 (NP175117.1)
A.thaliana Prx10 (NP175380.2)
A.thaliana Prx5 (NP172907.1)
A.thaliana Prx68 (NP200648.1)
A.thaliana Prx53 (NP196290.1)
A.thaliana Prx22 (NP181372.1)
A.thaliana Prx37 (NP192617.1)
A.thaliana Prx34 (NP190481.1)
A.thaliana Prx32 (NP850652.1)
A.thaliana Prx40 (NP193362.2)
OePOX17-like
S.lycopersicum peroxidase 17 (XP004250108.1)
S.indicum peroxidase 17 (XP011097539.1)
G.hirsutum gaiacol peroxidase (AF485266.1)
P.trichocarpa peroxidase 17 (XP002310551.1)
V.vinifera peroxidase 17 (XP002271083.1)
A.thaliana Prx17 (NP179828.1)
E.grandis peroxidase 17 (XP010025419.1)
C.saticus peroxidase 17-like (XP004164248.1)
C.arietinum peroxidase 17-like (XP004505514.1)
G.max peroxidase 17-like (XP003540551.1)
M.notabilis peroxidase 17 (XP010106276.1)
F.vesca peroxidase 17-like (XP004289301.1)
P.mume peroxidase 17-like (XP008235027.1)
M.domestica peroxidase 17 (XP008381982.1)
E.grandis peroxidase 64-like (XP010041794.1)
V.vinifera peroxidase 64 (XP003634364.1)
G.max peroxidase 64 (XP003545020.1)
C.sativus peroxidase 64-like (XP004143079.1)
P. bretschneideri peroxidase 64 (XP009363452.1)
M.domestica peroxidase 64 (XP008393813.1)
P.mume peroxidase 64 (XP008242033.1)
F.vesca peroxidase 64-like (XP004287777.1)
C.aritinum peroxidase 64-like (XP004509399.1)
R.communis peroxidase 64 (XP002531135.1)
OePOX64-like
S.indicum peroxidase 64-like (XP011078263.1)
S.lycopersicum peroxidase 64-like (XP004232515.1)
N.sylvestris peroxidase 64-like (XP009767397.1)
A.thaliana Prx64 (NP199033.1)
M.notabilis peroxidase 64 (XP010113299.1)
A.thaliana Prx12 (NP177313.1)
A.thaliana Prx51 (NP001078508.1)
A.thaliana Prx55 (NP196917.1)
C.arietinum peroxidase 55-like (XP004513315.1)
P.mume peroxidase 55 (XP008231106.1)
M.domestica peroxidase 55-like (XP008379105.1)
E.grandis peroxidase 51-like (XP010033422.1)
M.notabilis peroxidase 55 (XP010112376.1)
OePOX55-like
V.vinifera peroxidase 55-like (XP002274131.2)
F.vesca peroxidase 55-like (XP004303962.1)
G.arboreum peroxidase 51-like (KHG27040.1)
S.indicum peroxidase 51 (XP011097275.1)
C.sativus peroxidase 51-like (XP004166427.1)
N.sylvestris peroxidase 55-like (XP009802952.1)
S.lycopersicum peroxidase 51 (XP010315910.1)
A.thaliana Prx3 (NP172018.1)
A.thaliana Prx1 (NP563732.1)
E.grandis peroxidase 42-like (XP010057924.1)
P.tricocharpa peroxidase 42 (XP002304909.1)
S.indicum peroxidase 42-like (XP011096066.1)
A.marina secretory peroxidase (BAB16317.1)
C.roseus secretory peroxidase (AAY26520.1)
A.thaliana Prx42 (NP567641.1)
V.vinifera peroxidase 42 (XP002274769.1)
C.sativus peroxidase 42-like (XP004165387.1)
M.notabilis peroxidase 42 (XP010092206.1)
C.sinensis peroxidase 42-like (XP006467438.1)
P. bretschneideri peroxidase 42-like (XP009360637.1)
M.domestica peroxidase 42-like (XP008345825.1)
OePOX42-like
I.batatas swpb6 (AFY26877.1)
S.lycopersicum peroxidase 42-like (XP004232712.1)
N.sylvestris peroxidase 42-like (XP009779064.1)
A.thaliana Prx21 (NP181250.1)
A.thaliana Prx29 (NP566565.1)
0.2

Supplementary Figure S.5. Neighbour-joining tree generated using amino acid sequences for putative olive class III peroxidase and PRXs from others species. Bootstrap replicates were used to determine the level of support at each node. Accession numbers are indicated in brackets.

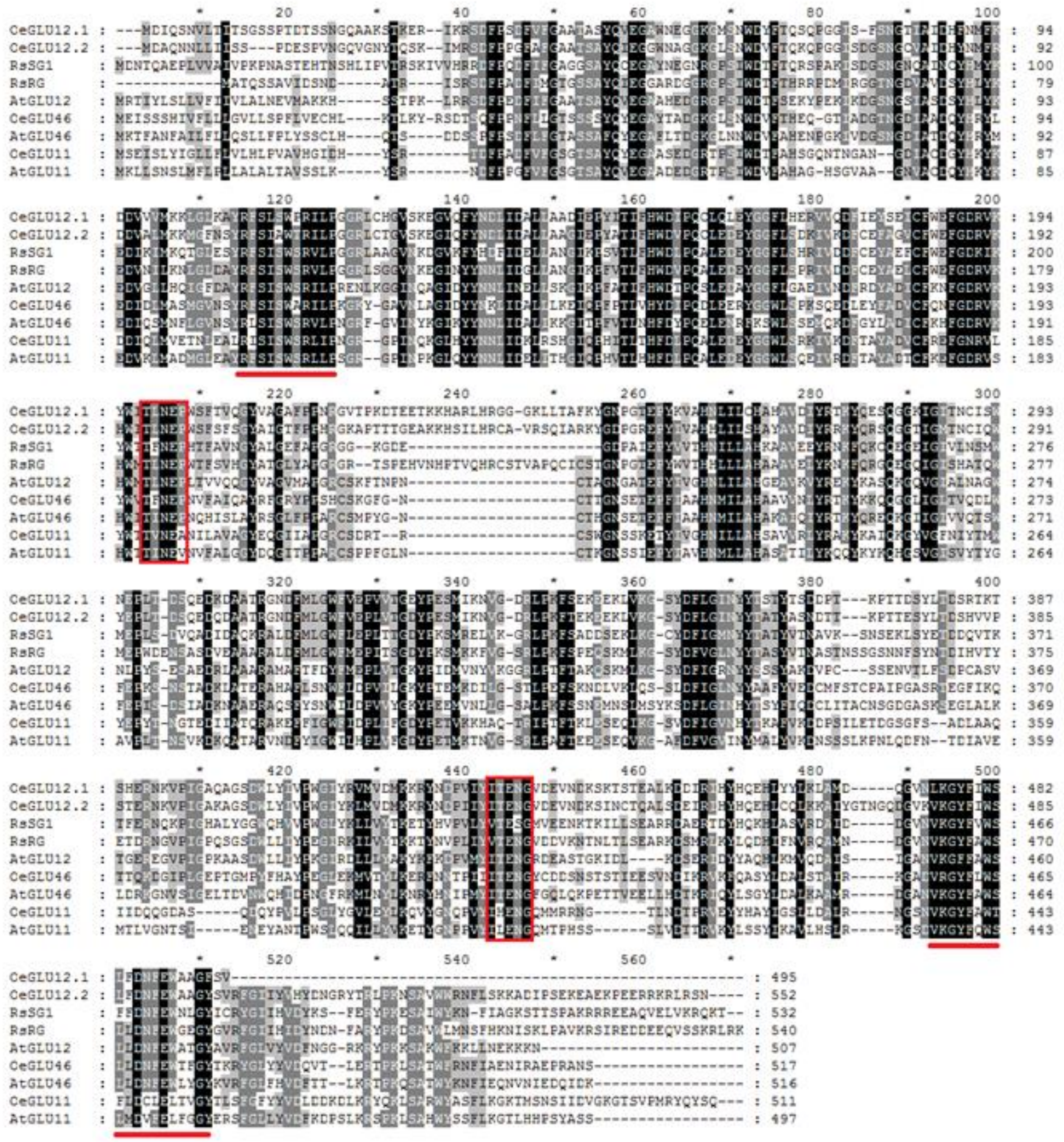

Supplementary Figure S.6. Sequence alignment of predicted olive GH1 family β-glucosidase proteins. The two glutamate residues within the conserved motifs TF/LNEP and I/VTENG or TVNEA/IHENG are indicated by red boxes. Other conserved GH1 family motifs, RFSIWSRIFP and GYFAWSLXDNFEW are highlighted in red.

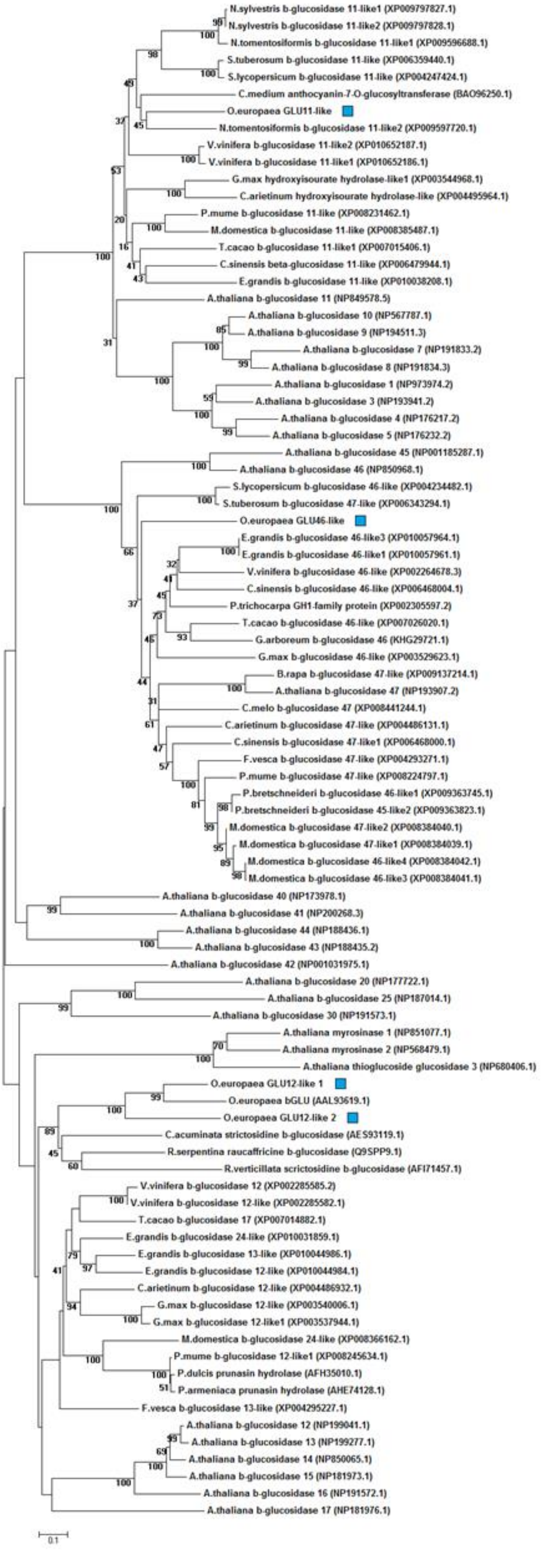
N.sylvestris b-glucosidase 11-like1 (XP009797827.1)
N.sylvestris b-glucosidase 11-like2 (XP009797828.1)
N.tomentosiformis b-glucosidase 11-like1 (XP009596688.1)
S.tuberosum b-glucosidase 11-like (XP006359440.1)
S.lycopersicum b-glucosidase 11-like (XP004247424.1)
C.medium anthocyanin-7-O-glucosyltransferase (BAO96250.1)
O.europaea GLU11-like
N.tomentosiformis b-glucosidase 11-like2 (XP009597720.1)
V.vinifera b-glucosidase 11-like2 (XP010652187.1)
V.vinifera b-glucosidase 11-like1 (XP010652186.1)
G.max hydroxyisourate hydrolase-like1 (XP003544968.1)
C.arietinum hydroxyisourate hydrolase-like (XP004495964.1)
P.mume b-glucosidase 11-like (XP008231462.1)
M.domestica b-glucosidase 11-like (XP008385487.1)
T.cacao b-glucosidase 11-like1 (XP007015406.1)
C.sinensis beta-glucosidase 11-like (XP006479944.1)
E.grandis b-glucosidase 11-like (XP010038208.1)
A.thaliana b-glucosidase 11 (NP849578.5)
A.thaliana b-glucosidase 10 (NP567787.1)
A.thaliana b-glucosidase 9 (NP194511.3)
A.thaliana b-glucosidase 7 (NP191833.2)
A.thaliana b-glucosidase 8 (NP191834.3)
A.thaliana b-glucosidase 1 (NP973974.2)
A.thaliana b-glucosidase 3 (NP193941.2)
A.thaliana b-glucosidase 4 (NP176217.2)
A.thaliana b-glucosidase 5 (NP176232.2)
A.thaliana b-glucosidase 45 (NP001185287.1)
A.thaliana b-glucosidase 46 (NP850968.1)
S.lycopersicum b-glucosidase 46-like (XP004234482.1)
S.tuberosum b-glucosidase 47-like (XP006343294.1)
O.europaea GLU46-like
E.grandis b-glucosidase 46-like3 (XP010057964.1)
E.grandis b-glucosidase 46-like1 (XP010057961.1)
V.vinifera b-glucosidase 46-like (XP002264678.3)
C.sinensis b-glucosidase 46-like (XP006468004.1)
P.trichocarpa GH1-family protein (XP002305597.2)
T.cacao b-glucosidase 46-like (XP007026020.1)
G.arboreum b-glucosidase 46 (KHG29721.1)
G.max b-glucosidase 46-like (XP003529623.1)
B.rapa b-glucosidase 47-like (XP009137214.1)
A.thaliana b-glucosidase 47 (NP193907.2)
C.melo b-glucosidase 47 (XP008441244.1)
C.arietinum b-glucosidase 47-like (XP004486131.1)
C.sinensis b-glucosidase 47-like1 (XP006468000.1)
F.vesca b-glucosidase 47-like (XP004293271.1)
P.mume b-glucosidase 47-like (XP008224797.1)
P.bretschneideri b-glucosidase 46-like1 (XP009363745.1)
P.bretschneideri b-glucosidase 45-like2 (XP009363823.1)
M.domestica b-glucosidase 47-like2 (XP008384040.1)
M.domestica b-glucosidase 47-like1 (XP008384039.1)
M.domestica b-glucosidase 46-like4 (XP008384042.1)
M.domestica b-glucosidase 46-like3 (XP008384041.1)
A.thaliana b-glucosidase 40 (NP173978.1)
A.thaliana b-glucosidase 41 (NP200268.3)
A.thaliana b-glucosidase 44 (NP188436.1)
A.thaliana b-glucosidase 43 (NP188435.2)
A.thaliana b-glucosidase 42 (NP001031975.1)
A.thaliana b-glucosidase 20 (NP177722.1)
A.thaliana b-glucosidase 25 (NP187014.1)
A.thaliana b-glucosidase 30 (NP191573.1)
A.thaliana myrosinase 1 (NP851077.1)
A.thaliana myrosinase 2 (NP568479.1)
A.thaliana thioglucoside glucosidase 3 (NP680406.1)
O.europaea GLU12-like 1
O.europaea bGLU (AAL93619.1)
O.europaea GLU12-like 2
C.acuminata strictosidine b-glucosidase (AES93119.1)
R.serpentina raucaffricine b-glucosidase (Q9SPP9.1)
R.verticillata scrictosidine b-glucosidase (AFI71457.1)
V.vinifera b-glucosidase 12 (XP002285585.2)
V.vinifera b-glucosidase 12-like (XP002285582.1)
T.cacao b-glucosidase 17 (XP007014882.1)
E.grandis b-glucosidase 24-like (XP010031859.1)
E.grandis b-glucosidase 13-like (XP010044986.1)
E.grandis b-glucosidase 12-like (XP010044984.1)
C.arietinum b-glucosidase 12-like (XP004486932.1)
G.max b-glucosidase 12-like (XP003540006.1)
G.max b-glucosidase 12-like1 (XP003537944.1)
M.domestica b-glucosidase 24-like (XP008366162.1)
P.mume b-glucosidase 12-like1 (XP008245634.1)
P.dulcis prunasin hydrolase (AFH35010.1)
P.armeniaca prunasin hydrolase (AHE74128.1)
F.vesca b-glucosidase 13-like (XP004295227.1)
A.thaliana b-glucosidase 12 (NP199041.1)
A.thaliana b-glucosidase 13 (NP199277.1)
A.thaliana b-glucosidase 14 (NP850065.1)
A.thaliana b-glucosidase 15 (NP181973.1)
A.thaliana b-glucosidase 16 (NP191572.1)
A.thaliana b-glucosidase 17 (NP181976.1)
0.1

Supplementary Figure S.7. Neighbour-joining tree generated using amino acid sequences for putative Oeβ-GLU from others species. Bootstrap replicates were used to determine the level of support at each node. Accession numbers are indicated in brackets.
